# Artificial intelligence assisted diagnosis of early tc markers and its application

**DOI:** 10.1007/s12672-024-01017-w

**Published:** 2024-05-18

**Authors:** Laney Zhang, Chinting Wong, Yungeng Li, Tianyi Huang, Jiawen Wang, Chenghe Lin

**Affiliations:** 1 Yale School of Public Health, New Haven, CT USA; 2 Department of Nuclear Medicine, The First Hospital of Jilin University, Changchun, Jilin China; 3 University College London, London, UK

**Keywords:** Thyroid cancer, Artificial intelligence, Auxiliary diagnosis

## Abstract

Thyroid cancer (TC) is a common endocrine malignancy with an increasing incidence worldwide. Early diagnosis is particularly important for TC patients, because it allows patients to receive treatment as early as possible. Artificial intelligence (AI) provides great advantages for complex healthcare systems by analyzing big data based on machine learning. Nowadays, AI is widely used in the early diagnosis of cancer such as TC. Ultrasound detection and fine needle aspiration biopsy are the main methods for early diagnosis of TC. AI has been widely used in the detection of malignancy in thyroid nodules by ultrasound images, cytopathology images and molecular markers. It shows great potential in auxiliary medical diagnosis. The latest clinical trial has shown that the performance of AI models matches with the diagnostic efficiency of experienced clinicians, and more efficient AI tools will be developed in the future. Therefore, in this review, we summarized the recent advances in the application of AI algorithms in assessing the risk of malignancy in thyroid nodules. The objective of this review was to provide a data base for the clinical use of AI-assisted diagnosis in TC, as well as to provide new ideas for the next generation of AI-assisted diagnosis in TC.

## Introduction

Thyroid cancer (TC), as the most prevalent endocrine malignancy worldwide, accounts for 3.4% of cancers diagnosed each year [1]. In the report on cancer incidence in 2020 published by the International Agency for Research on Cancer (IARC), the incidence of TC ranked the ninth among cancers [2]. Although both men and women may suffer from TC, women account for three quarters of all TC sufferers. Additionally, there is no significant age limit for the incidence of TC. Briefly, elderly TC patients are more common, but the detection rate of TC in young and adult patients aged 16–33 years is higher [3]. As shown in a previous study, there were 43,000 new cases of TC and 2230 TC-caused deaths in the United States in 2022 [4]. Currently, the primary clinical diagnostic approach for TC is still palpation, which accounts for approximately 30–40% of TC diagnosis. Ultrasound or other imaging techniques are the primary imaging modality for patients with nonpalpable thyroid nodules to identify the number and characteristics of the nodules and the high-risk features associated with increased risk of malignancy [5]. Fine needle aspiration (FNA) is used to further determine the risk of thyroid nodules through biopsy [6]. Although the mortality rate of patients with TC is relatively low, a series of problems arising from the over-diagnosis of TC should not be underestimated. Over-diagnosis leads to unnecessary surgeries featured with potential complications, unnecessary personal and social treatment cost, adverse psychological impact of the cancer diagnosis on the patients, and a significant decrease in the quality of life of the patients diagnosed [7]. Although clinicians are currently trying to avoid over-diagnosis, it is difficult to strike a balance. After all, not all patients with progressive and metastatic TC have palpable thyroid nodules, and not all characteristics of TC with the lowest risk are clear [8]. Correct early diagnosis is conductive to the treatment of patients with TC. Therefore, it is necessary to improve the current diagnostic strategies to increase the accuracy of diagnosis.

Artificial intelligence (AI), belonging to the field of computer science, is dedicated to building intelligent machines capable of performing tasks that typically require human-level intelligence. AI is defined as a programmed machine that can learn and recognize the patterns and relationships between inputs and outputs, and can effectively utilize such knowledge to make a decision based on brand-new input data [9]. AI is mainly composed of two virtual branches: machine learning and deep learning. The algorithms of deep learning can also be categorized into supervised, unsupervised and reinforcement learning. Among them, the most commonly understood deep learning scheme is the convolutional neural network (CNN), which represents a special type of multilayer artificial neural network and is very effective for image classification [10]. In the last decade, with the advancement of big data, algorithms, computer power and Internet technologies, deep learning has achieved unprecedented success in a variety of tasks in various fields, including facial recognition, image classification, speech recognition, automatic translation, and healthcare [11]. AI has attracted considerable attention in various medical fields, and the Food and Drug Administration (FDA) also has approved many AI algorithms related to oncology [12]. In 2017, a precision medicine system using AI as an aid to diagnosis was gradually established in China [13]. As described by a previous study, AI effectively improved uneven allocation of medical resources, reduced medical costs, and increased medical efficiency. Li, X., et al. indicated that in the diagnosis of TC, the specificity of AI-assisted diagnosis and treatment was higher than that of three experienced radiologists [14]. Hence, AI-assisted therapy has the potential to improve the diagnosis of TC. This review provided an in-depth understanding and analysis of the application of AI in the diagnosis and treatment of TC based on the existing literature. Also, we explored the role of AI systems in recent advance in medical diagnosis and treatment, thereby providing new insights into AI and TC research.

### Search strategy

Regarding the application of AI in TC diagnosis, we have systematically reviewed and studied existing literature to assess the potential and efficacy of AI technologies in improving diagnostic accuracy and enhancing patient experience. These applications of AI demonstrate its expanding footprint within the healthcare sector. To systematically understand the developments in this field, our review adopts a structured literature search strategy. The following sections will detail our search methods and criteria for study selection:

(1) Database selection: We conducted literature searches through websites such as PubMed, Web of Science and IEEE Xplore. (2) Keywords and search terms: Artificial Intelligence (AI-assisted; Machine Learning; Deep Learning), Thyroid Cancer (Thyroid Nodule; Cancer Diagnosis). (3) Exporting and managing search results: We exported the literature to EndNote reference manager and organized the literature using folders, tags, and annotations for quick retrieval. (4) Inclusion and exclusion criteria: inclusion criteria, studies involving the application of AI in TC diagnosis; articles including reviews, case-control studies, or technical assessment reports; articles providing sufficient data to evaluate the diagnostic performance of AI technology; Publication date from January 2015 to April 2024. Exclusion criteria: non-English articles; studies lacking sufficient methodological quality or incomplete data.

### Early diagnosis of thyroid cancer

According to a current study, TC originates from parafollicular cells or follicular epithelial cells. TC derived from follicular cells are often categorized into 4 histological types, namely, papillary thyroid carcinoma, follicular thyroid carcinoma (FTC), poorly differentiated thyroid carcinoma, and anaplastic TC. Papillary thyroid carcinoma is the most common histological type, accounting for 80%–85% of all patients with TC, whereas both poorly differentiated thyroid carcinoma and anaplastic TC account for no more than 2%. Despite diverse TC types, they can all be relieved and improved through early diagnosis and treatment [15]. Therefore, early diagnosis is particularly important for different types of TC.

In the early stage, TC mainly exists in the form of thyroid nodules. Data showed that TC occurred in 7–15% of thyroid nodules, thus it is particularly essential to accurately differentiate benign or malignant nodules for the early diagnosis of TC [16].

In the conventional imaging tests, the risk of developing into TC or malignancy is determined by observing the composition, shape, echogenicity, margins, calcification, and echogenic foci of the TC [17]. In addition, with the progression of research, the risk factors, such as gender, age, associated with the development of thyroid nodules into cancer have been revealed [18]. Wang, Z., et al. indicated that the number of female patients was high, while the probability of thyroid nodule worsening in male patients was 1.6 times higher than that in female patients [19]. Similarly, although the prevalence of thyroid nodules increases with age, the risk of cancerous thyroid nodules decreases with age. Besides, young patients are more likely to suffer from the worsening nodules than old patients. Hence, the results of ultrasound examination need to be perfected by other risk factors during the clinical diagnosis of thyroid nodules [20]. Notably, the results of ultrasonography alone are not reliable, so other diagnostic methods need to be supplemented.

In addition to ultrasonography, FNA is an essential tool applied in the diagnosis of most patients with thyroid nodules, which primarily assesses the risk of TC through cytologic analysis [21]. FNA is currently considered the gold standard for preoperative diagnosis of thyroid nodules. When the nodules can not be clearly judged by ultrasound, FNA can be employed to further determine the condition of the nodules and the risk of cancer at the cellular level [22]. Although FNA contributes to differentiating benign and malignant thyroid nodules, it may cause pain and small hematomas in patients. In addition, FNA cannot distinguish adenomas with follicular lesions from cancer [23]. All in all, ultrasonography and FNA are still the most widely used clinical examinations. Comparatively speaking, the use of ultrasonography is more common. However, various hospitals or agencies may employ different diagnostic criteria for thyroid nodules, which also affects the accurate judgment of the disease. Therefore, it is necessary to standardize and unify the criteria and accuracy of early diagnosis of TC.

### Markers for early diagnosis of thyroid cancer

The characteristics of thyroid nodules, including the composition, shape, echogenicity, margins, calcification and echogenicity, are often used as indicators of deterioration in ultrasonography [24]. However, the specificity of these indicators is relatively poor. A solid and hypoechoic nodule with an irregular margin is defined as malignancy based on the standard of ultrasonography. Nevertheless, benign patients may also suffer from solid tumor. Not all hypoechoic nodules are malignant tumors, and malignant tumors may also exist at regular margins [25]. Liu et al. observed that advanced TC was characterized by rough and dispersed margins. In their study, there were 56 benign thyroid nodule patients with smooth margins, accounting for 70% of all benign patients; besides, there were 48 malignant nodule patients with smooth margins, accounting for 30% of all malignant patients. Additionally, of patients with non-smooth margins, the number of patients with benign nodules was 24, accounting for 30% of all patients with benign nodules; while that with malignant nodules was 112, accounting for 70% of all patients with malignant nodules [26]. These manifestations can not be taken as the only criteria, and further diagnosis is required.

Clinically, although patients are diagnosed with TC by ultrasound, other molecular markers are still needed to improve the accuracy of diagnosis. A study suggested that, because elevated serum thyroglobulin (Tg) can be seen in patients with benign thyroid disease, the measurement of Tg is helpful to determine the prognosis of patients receiving differentiated TC treatment [27]. However, Haugen, B.R., et al., did not recommend to assess the prognosis of patients undergoing thyroidectomy using Tg level only, as elevated Tg might also be a manifestation of the development of other cancers [28]. In the past two decades, the non-coding RNA has played a decisive role in gene expression as a key regulator [29]. Besides, a number of studies have demonstrated that non-coding RNAs, especially microRNAs (miRNAs), circular RNAs (circRNAs) and long non-coding RNAs (lncRNAs), play a key role in the occurrence and development of TC [30]. MiRNAs are short non-coding RNA molecules with a length of approximately 22 nucleotides. They regulate gene expression at the post-transcriptional level by binding to complementary sequences on target mRNAs, thereby influencing mRNA stability and translation efficiency. Aberrant miRNA expression has been associated with the development of various diseases such as TC [31]. As shown in a study by Zhao, L., X. Zhang, and S. Cui, miR-21 was not only highly expressed in the tissues of medullary thyroid carcinoma but also could bind to and inhibit the expression level of programmed cell death protein 4 (a tumor suppressor) [32]. Besides, circRNAs have a closed-loop structure that grants them enhanced stability and a range of biological functions. In the onset and progression of TC, circRNAs play roles in disease progression through mechanisms such as acting as miRNA sponges, modulating mRNA expression, and regulating protein function [33]. Compared to normal tissues, there were 54 differentially expressed circRNAs in TC tissues, of which 19 showed a down-regulation trend, and 35 were up-regulated [34]. LncRNAs are RNA molecules with a length exceeding 200 nucleotides, playing roles in maintaining cellular structure and intracellular signal transduction [35]. Similarly, more and more lncRNAs have been identified to regulate the proliferation, migration, invasion and other malignant biological properties of TC [36]. It can be seen that non-coding RNAs show great potential for the diagnosis of TC. However, the results of the existing studies are not sufficient to develop diagnostic strategies regarding non-coding RNAs, as the dysregulated expression of these non-coding RNAs is not the only molecular event in the progression of TC. Moreover, there are still many potentially critical non-coding RNAs that have not been revealed.

### AI-assisted diagnosis

In addition to traditional clinical diagnostic methods, Computer Aided Detection or Diagnosis (CAD) systems are becoming more and more common [14]. CAD combines AI with radiology and pathology image processing to assist the detection and diagnosis of disease and to reduce the time required for image parsing [37]. Currently, AI has been widely used in the field of medical imaging such as radiography [38], ultrasonography [39, 40], and case image diagnosis [41]. Overall, AI has great potential in assisting medical diagnosis.

The efficiency of AI-assisted diagnosis continues to be improved, which opens up new prospects for cancer diagnosis and staging modalities [42]. Among them, the whole slide image provides the basis of AI-assisted diagnosis in pathology detection [43]. In addition, the image data from ultrasound testing are the important basis for AI-assisted diagnosis. On the one hand, AI can extract multiple features using computer vision algorithms for diagnosis and prediction. On the other hand, AI can provide accurate and objective assessment of immunohistochemical biomarkers (e.g., Ki67, PD-L1), quantification of cells, and evaluation of spatial arrangement, expression, density, and distribution patterns of cells [44]. Moreover, AI can be used to detect isolated tumor cells in lymph nodes suspected to be metastatic cancer, increasing detection sensitivity in a time-saving manner [45]. Another high-profile feature of AI is content-based image retrieval, which enables pathologists to search for related images from repositories of large histopathology databases. Such a feature is particularly important to guide pathologists in diagnosing rare and complex cases that they may occasionally encounter in clinical practice. The images searched from databases reflected the similarities of the relevant histopathologic features, not just image similarities [46, 47]. Therefore, AI has been widely applied in the diagnosis of cancers, including TC. Machine learning and deep learning techniques can assist in analyzing ultrasound results and biopsy samples for the prediction, diagnosis, and treatment of TC.

### Operational mechanisms of AI-assisted thyroid diagnosis

The working principle of AI-assisted diagnosis for TC involves analyzing vast amounts of data to learn the distinguishing features between benign and malignant lesions [48]. The specific workflow and principles underlying AI-assisted TC diagnosis are as follows: (1) Data collection and processing: involves gathering pathological slice images of thyroid tissues, ultrasound images from thyroid examinations, and clinical data such as thyroid function test results, gene and protein expression data, among others. Subsequently, the data undergo standardization and normalization. (2) Feature extraction: features extracted include the size, shape, and boundaries of nodules, internal echo patterns within nodules, blood flow distribution, and others. (3) Machine learning algorithm training: Utilizing machine learning algorithms, such as CNN, the AI model is trained. The model identifies patterns and differences between benign and malignant lesions by learning from a large dataset with labels. (4) Validation and testing: The AI model is evaluated on an independent testing dataset to validate the reliability. (5) Clinical integration: integrating the AI model into clinical workflows as an auxiliary tool enables physicians to enhance diagnostic accuracy [49]. Over time and with the accumulation of more data, the AI model may require periodic updates to maintain or improve its performance.

### AI-assisted tools for diagnosing thyroid cancer

The deep combination of AI and medical technology is attributed to improving the accurate treatment of TC, increasing the speed of diagnosis, avoiding the bias caused by subjective factors of doctors, and providing more accurate and effective treatments for individual patients [50]. Thyroid tumor detection relies on medical imaging analysis such as, ultrasound imaging [51], magnetic resonance imaging [52], and computed tomography [53]. Ultrasound detection possesses safe and noninvasive properties and can provide good visualization of the mass, so it acts as the preferred and most used method for initial screening and diagnosis of thyroid nodules. By far, a number of AI tools have been approved by the Food and Drug Administration (FDA). S-Detect 2, as a system based on deep learning developed in 2020, can be used for real-time diagnosis of thyroid nodules. Briefly, when a nodule is identified during the scanning process, the image is frozen and a square area is manually drawn around the nodule. The edge of the nodule is then automatically segmented, followed by the collection of information on the main features of the nodule (composition, orientation, echo, shape, boundaries) and the risk stratification (benign or malignant) [54]. AmCAD-UT, developed in 2018, is a Microsoft Windows-based software that can analyze the static images of thyroid nodules after image acquisition. However, this program requires the operator to define the boundary of the nodule, and then can be applied in the collection of nodule-specific information and the risk assessment [55]. Koios DS is the latest AI-assisted diagnostic tool for TC approved by the FDA. Similar to AmCAD-UT, Koios DS is a software integrated into the radiology workstation. The operator is still required to autonomously select two static orthogonal images for the nodule and create a square area around the nodule to determine the area to be analyzed. Notably, Koios DS takes less than 1 s to analyze the nodule [56]. Medo Thyroid is an FDA-approved tool for automated segmentation of the thyroid gland and thyroid nodules, which is highly consistent with measurements taken by experienced radiologists in extracting the thyroid gland and nodules [57]. The AI tools listed in this paper are only a portion of those utilized in the diagnosis of thyroid nodules, and there are certainly quite a few more tools emerging at the time of this writing. Therefore, it can be predicted that more and more tools will be developed and utilized in the diagnosis of TC.

### AI-assisted ultrasound for thyroid cancer diagnosis

The echo of thyroid nodules can be divided into hypoechoic, isoechoic and hyperechoic. Previous studies have shown that hypoechoic nodules with irregular borders are more likely to develop into malignant nodules. Since the textural features of ultrasound images are complex and the visual appearance of malignant and benign nodules is similar, the recognition of ultrasound images is highly dependent on the experience of doctors. It takes a long time for clinicians to master ultrasound detection and the influence of subjective factors on the diagnosis is difficult to eliminated Accurate segmentation of thyroid nodule regions by ultrasound is a prerequisite for the diagnosis of TC. The VGG-16-based deep learning model is applied in automatic detection and segmentation of thyroid nodule images in medicine [58]. Yufan Chen et al. developed a combination of the American College of Radiology Thyroid Imaging Reporting and Data System and AI multitasking deep learning for evaluating benign and malignant thyroid nodules [39]. The guidance of super-resolution reconstruction network can make the high-frequency information of thyroid ultrasound images more comprehensive and abundant than the original images, and the proposed ultrasound image segmentation based on this can provide reliable auxiliary diagnostic information for doctors in clinical practice [59]. Antonin Prochazka et al. detected thyroid nodules using a histogram analysis and a segmentation-based fractal texture analysis algorithm. Then, they further differentiated the benign and malignantnodules through support vector machine and random forests classifiers [60]. As reported in a study, deep learning combined with important ultrasound features can achieve similar or better diagnostic performance for thyroid nodules than experienced radiologists, and such diagnostic efficiency can often reach or exceed the level of radiologists [61]. Based on the deep CNN model, the diagnostic accuracy of TC can be improved by analyzing ultrasound imaging data [34]. Li et al. demonstrated that AI-assisted diagnosis had similar sensitivity and higher specificity compared to experienced radiologists in identifying TC [14]. The study by Jin et al. re-emphasized the importance of AI-assisted diagnosis. Briefly, compared with experienced radiologists, deep learning achieved higher accuracy in diagnosing TC under the guidance of ultrasound feature [62]. Some other studies have shown that with the help of AI, radiologists can improve their ability to identify features such as irregular margins, extra-thyroidal extension, large comet tail artifacts, large calcifications, peripheral calcifications, and punctate echogenic lesions [63, 64]. The above studies have suggested that AI-assisted diagnosis can improve the accuracy of ultrasound diagnosis of TC. Fatemeh Abdolali et al. proposed a nodule detection approach based on a deep neural network architecture and reduced the complex subsequent processing steps through the designed loss function regularization and network hyperparameters [57].

At present, the commonly used algorithm to assist ultrasound diagnosis is S-Detect, which is a program integrated into an ultrasound machine to semi-automatically detect and classify thyroid lesions. In a prospective study, researchers collected the images of 4,919 nodules to train the initial S-Detect system, and subsequently validated this system prospectively on 286 thyroid nodules. The negative predictive value of this algorithm was 88.1% and the positive predictive value was 84.5% [65]. Jeong, E.Y., et al. demonstrated that utilizing AI algorithms could increase the diagnostic efficiency of radiologists who lacked the test of nodules, and could increase the sensitivity, specificity, positive predictive value, and negative predictive value from 68.3%, 74.1%, 68.9% and 72.7% to 73.8%, 88.5%, 73.3% and 80.0%, respectively [66]. Dat Tien Nguyen et al. used weighted binary cross-entropy loss function for the training of deep CNN and proposed an AI-assisted ultrasound image method for diagnosis of malignant thyroid nodule based on spatial and frequency domain analysis. The experience dependence of diagnosis by a doctor could be reduced via computer-assisted diagnosis [67]. Meanwhile, in the results of a meta-analysis by Xu, L., et al., it could be observed that CAD systems based on deep learning all had good performance in diagnosing malignant thyroid nodules compared to classical machine learning. However, the study concluded that the diagnostic performance of AI algorithms based on deep learning was comparable to that of radiologists, and the difference was not significant. In addition, they proposed that the experienced radiologists may still have an advantage over AI algorithms in the real-time diagnostic process [68]. In the meta-analysis by Yu et al. patients were grouped by age, and the analysis results showed that AI-assisted diagnostic techniques had higher diagnostic performance in subjects with an average age of < 50 years [69]. However, it is still important to note that benign nodules detected by ultrasound are likely to be histopathologically malignant, so the AI algorithms should not be expected to be foolproof. Furthermore, improving the differentiation of benign and malignant thyroid nodules of category 4 and 5 through deep learning has important potential for clinical application [70]. Most AI models are trained on images of classical thyroid nodules. In fact, only a few images involve follicular variation of TC, FTC and medullary thyroid carcinoma. Lu Tang et al. designed a texture and shape focused dual-stream attention neural network that could enhance the extraction of shape features and improve the classification performance [71]. Most of the previous studies have compared the diagnostic effectiveness of TC between AI-assisted diagnosis and radiologists, but fewer have observed their combination. It is not difficult to speculate from the above results that the combination may have a better effect.

### AI-assisted fine needle aspiration biopsy for the diagnosis of thyroid cancer

Suspicious thyroid nodules detected by ultrasonography are usually further examined by FNA [72], and AI can assist FNA in the diagnosis of TC. One study showed that with the assistance of AI, the diagnostic sensitivity and specificity of FNA to goiter and lymphocytic thyroiditis were 90.48% and 83.33%, respectively [73]. In addition, the sensitivity and specificity of the AI algorithm established by Guan et al. to the diagnosis of malignant nodules were as high as 100% and 94.91% [74]. Notably, FNA is not sensitive to determining benign and malignant follicular lesions [75]. However, Shapiro and his colleagues utilized AI algorithms of cytology, morphology, and image to judge follicular lesions based on Giemsa-stained pathologic images The accuracy of these three algorithms was 93%, 96%, and 87%, respectively, which were significantly higher than that of FNA detection alone [76]. Therefore, under the synergy of AI-assisted diagnosis, the shortcomings of FNA in the current diagnosis of TC are expected to be improved.

However, AI-assisted diagnosis also requires manual segmentation of the region of interest, which may overlook important information. To address these shortcomings, a variety of new algorithmic systems have been built. Elliott Range et al. designed a two-step fully automated classification system that used the whole slide image to predict malignant tumors. In the first step, CNN automatically selected a region of interest containing follicular cells. In the second step, the pathology in the targeted region was detected and calculated by CNN. They used 799 pathology images to train the CNN model and tested the trained CNN model on 109 pathology images. In their results, the Area Under Curve diagnosed by the CNN model was 0.931, similar to that diagnosed by cytopathologists (0.932); besides, the specificity was 90.5% and the sensitivity was 92.0% [77]. Furthermore, Margari et al. constructed two cytologic diagnosis prediction models based on classification and regression trees (CARTs) models. The CART-C model was adopted to predict cytologic diagnosis, and the CART-H model to predict benign and malignant histologic diagnosis. The CART-C model had similar sensitivity, specificity, and accuracy compared to cytologic diagnosis by pathologists; while the CART-H model had higher specificity (93.63%), sensitivity (92.43%), and accuracy (93.03) than those of cytological diagnosis [78]. A text- and semantics-based semantic analysis support vector machine (SVM) model was used by Maleki et al. to analyze microscopic descriptions on cytopathology reports to differentiate non-invasive follicular thyroid neoplasm with papillarylike nuclear features from classical TC. The research reported an area under curve of 0.76, a sensitivity of 72.6%, and a specificity of 81.6% [79]. All of the above studies have shown that AI is more convenient for cytologic diagnosis. However, due to the lack of equipment and the high cost of patient data storage, the application of AI in cytopathology is limited. Fortunately, new algorithms will break through this limitation in the near future.

### AI-assisted molecular diagnosis of thyroid cancer

Clinically, when TC cannot be identified by ultrasound or FNA, molecular testing will be initiated [80], including the measurement of DNA mutations [81]. Previous tests of cancer-associated DNA mutation have focused only on the DNA mutation types with high prevalence but ignored the rare variants, thereby leading to misdiagnosis [82]. The AI algorithm (Afirma GSC) was used to determine the malignant status of TC based on the BRAF V600E mutation. The positive percent agreement of such a diagnostic method was 90.4%, while the negative percent agreement was 99.0% and the specificity was as high as 100% [83]. Hao et al. refined the analytical sensitivity for different amounts of RNA input and evaluated the analytical specificity for blood genomic DNA and other potentially interfering substances. The conclusion was that the Afirma Gene Sequencing Classifier (GSC) was tolerant to both RNA inputs in the range of 5–30 ng and 75% of test samples [84]. These results indicate that the stability and reproducibility of the AI-assisted algorithm analyses support their routine clinical application in thyroid nodules with indeterminate FNA cytology. A prospective study was conducted by Steward and his colleagues, in which they evaluated the diagnostic accuracy of an AI algorithm (ThyroSeq v3) for thyroid nodules with indeterminate cytology. The sensitivity and specificity of their study were 94% and 82%, respectively, with a negative predictive value of 97% and a positive predictive value of 66% for TC and invasive FTC with nuclei. In addition, they believed that the use of ThyroSeq v3 for auxiliary diagnosis could save up to 82% of patients with benign nodules of indeterminate cytology from diagnostic surgery [85]. Endo et al. compared two AI algorithms, Afirma Gene Expression Classifier (GEC) and GSC. Their outcomes revealed that, compared to GEC, the benign detection rate, positive predictive value, and characteristics of GSC were all significantly higher. Moreover, they observed that the surgical intervention rate for the thyroid nodule cohort with indeterminate cytology based on GSC was 17.6%, while that based on GEC was 52.5% [86]. It can be seen that as AI algorithms advance, they can provide more and more help for the diagnosis of TC. Interestingly, a study comparing the diagnostic efficiency of Afirma and Thyroseq for thyroid nodules with indeterminate cytology also confirmed that cytologically indeterminate nodules were more classified as benign by GSC compared with Afirma GEC; moreover, GSC had higher specificity and positive predictive value than Afirma GEC. Meanwhile, it was noted that Afirma GEC and Thyroseq had similar specificity and positive predictive value for the diagnosis of thyroid nodule [87]. Collectively, AI algorithms can increase the molecular diagnosis efficiency of thyroid nodules with indeterminate cytology and avoid secondary harm to patients due to unnecessary interventional diagnostic methods.

## Limitations

In the clinical practice of AI-assisted TC diagnosis, due to limited relevant research and experience, there are still challenges in practical application that require further study and exploration [49]. (1) Poor sample quality: inadequate sample quality can adversely affect the accuracy of AI-assisted diagnosis. (2) Immature analytical techniques: issues such as weak generalization ability of models and lack of standardized evaluation criteria highlight the immaturity of current analytical techniques. (3) Need for enhanced professional training of medical personnel: it is essential to provide appropriate AI technology training to enable them to effectively use and comprehend AI tools. (4) Improving patient acceptance: concerns regarding public acceptance of AI technology and trust in AI diagnostic recommendations warrant attention [88]. We believe that addressing these challenges will contribute to the successful implementation and adoption of AI technologies in TC diagnosis.

### Future and outlook

Currently, AI-assisted diagnosis for TC can not be fully automated based on the data from ultrasound, FNA, or molecular testing. With the rapid development of AI in the future, the performance of AI may exceed the current levels, New tools for AI-assisted diagnosis of TC may continue to emerge, and existing tools will also continue to be improved. Additionally, a number of AI-assisted tools have been developed to determine markers at the same level as experienced physicians. However, most of the current research has focused on the comparison between AI-assisted tools and doctors but ignored the effect of their combination on the diagnosis of TC. Therefore, if we combine both organically in diagnosis, will it produce unexpected results? This issue deserves further exploration.

## Conclusion

AI has increasingly become an indispensable part in enhancing the diagnostic accuracy and efficiency of TC treatment, particularly in the early detection of malignant tumors. The integration of AI technologies, including machine learning, deep learning, and computer vision, showing promise in revolutionizing the standard diagnostic approaches for TC, which traditionally heavily rely on ultrasound, FNA, and molecular testing. This study provides a comprehensive analysis and discussion of the application, operational mechanisms, and technological principles of AI-assisted techniques in TC diagnosis. It further elucidates the standardized approaches of AI systems for TC assessment and diagnosis, demonstrating high sensitivity and specificity in identifying malignant thyroid nodules, which offers a more holistic understanding for professionals in the related field.

Additionally, our study also offers a novel perspective to analyze the future directions and prospects of AI-assisted diagnosis. The ongoing development of AI tools can not only refine existing diagnostic models but also seamlessly integrate with emerging technologies such as genomics for comprehensive risk assessment and personalized treatment planning.

With the advancement of AI technologies, they offer immense potential for improving the diagnostic pathways for TC by enhancing accuracy, consistency, and efficiency. These advancements can lead to better prognostic outcomes through earlier and more precise therapeutic interventions, and play a pivotal role in clinical practice.

## Data Availability

The datasets used and/or analyzed during the current study are available from the corresponding author on reasonable request.
